# New Beginnings for mothers and babies in prison: A cluster randomized controlled trial

**DOI:** 10.1080/14616734.2013.782651

**Published:** 2013-04-04

**Authors:** Michelle Sleed, Tessa Baradon, Peter Fonagy

**Affiliations:** a Research Department of Clinical, Educational and Health Psychology, University College London, London, UK; b The Anna Freud Centre, London, UK

**Keywords:** prison, incarceration, infant, mother, attachment, intervention

## Abstract

Mothers in prison represent a high-risk parenting population. New Beginnings is an attachment-based group intervention designed specifically for mothers and babies in prison. This cluster randomized trial examined the outcomes for 88 mothers and babies participating in the New Beginnings program and 75 dyads residing in prisons where the intervention did not take place. Outcomes were measured in terms of parental reflective functioning, the quality of parent–infant interaction, maternal depression, and maternal representations. Mothers in the control group deteriorated in their level of reflective functioning and behavioral interaction with their babies over time, whereas the mothers in the intervention group did not. There were no significant group effects on levels of maternal depression or mothers' self-reported representations of their babies over time. An attachment-based intervention may mitigate some of the risks to the quality of the parent–infant relationship for these dyads.

## Introduction

The number of women serving prison sentences has increased dramatically worldwide over the last decade (Walmsley, 2012) resulting in increasing numbers of young children who are affected by maternal incarceration. It has been widely recognized that incarcerated mothers and their children represent a high-risk group. Many women in prison have been exposed to or are currently experiencing a number of difficulties which may impinge on their children's social and emotional development directly or indirectly. These include poor education, homelessness, and domestic violence (Prison Reform Trust, 2012), mental health problems (Birmingham, Coulson, Mullee, Kamal, & Gregoire, 2006; Gregoire, Dolan, Birmingham, Mullee, & Coulson, 2010), and drug and alcohol misuse (Singleton, Farrell, & Meltzer, 1997). A substantial proportion of adults in prison have experienced severe childhood trauma (Zlotnick, 1997), local authority care as a child, and previous sexual abuse (Prison Reform Trust, 2012). It is therefore unsurprising that a disproportionate number of mothers in prison are classified as insecure or unresolved with respect to loss or trauma in their attachment representations of their childhood relationships (Borelli, Goshin, Joestl, Clark, & Byrne, 2010). Many incarcerated adults have also been found to have a great deal of difficulty in their capacity for mentalization with regard to their own childhood attachment relationships (Levinson & Fonagy, 2004) and in thinking about their current relationship with their children (Baradon, Fonagy, Bland, Lenard, & Sleed, 2008). Mentalizing, the capacity to understand behavior in terms of underlying mental states, is thought to be pivotal to the intergenerational transmission of attachment patterns (Fonagy & Target, 2005; Slade, Grienenberger, Bernbach, Levy, & Locker, 2005) and the breakdown of this process in the parent has been implicated in a poor outcomes for the child (Sharp & Fonagy, 2008).

Multiple adverse outcomes have been reported for children whose mothers have served custodial sentences. These include antisocial-delinquent behavior, mental health problems, unemployment, school failure, drug abuse, and offending (Murray & Farrington, 2008).

### Infants co-residing with their mothers in prison

In the 1960s, Mother and Baby Units (MBUs) were formally established in UK prisons in recognition that in many cases the most favorable place for a young baby is with his or her mother, even when she is serving a custodial sentence (HM Prison Service, 2008). There are currently seven specialized MBUs in England and Wales where babies aged up to 18 months may remain with their mothers.

Permitting incarcerated mothers and their babies to remain together may prevent a cascade of devastating effects that may otherwise ensue from enforced separation and broken mother–infant attachments. One study has compared long-term outcomes for infants who remained with their mothers in a prison nursery versus those who were separated during infancy due to maternal incarceration (Goshin, 2010). At preschool age, children who co-resided with their mothers had fewer anxious/depressed behavioral problems than the separated infants, suggesting some long-term benefits of keeping mothers and babies together. However, both separated and co-residing infants were at risk of developing aggressive behavioral problems later on. Thus, infants who remain with their mothers in prison may still be impacted by the complex social and psychological difficulties that many of these families experience.

### The impact of the prison environment on the parent–infant relationship

The prison environment provides a complex array of factors which may have consequences for the mother–infant relationship. Some babies in MBUs have experienced separations from their mothers around the time of arrest, conviction, or while awaiting a decision of whether or not they will be allowed to stay together on a MBU. The separations can be sudden and sometimes families are not always able to prepare for them adequately (Murray & Murray, 2010). Some of the babies on MBUs will reach the maximum age allowed on the unit before their mothers' sentences are completed, and they may therefore face a separation from their mother in the future. Infants may also be affected by their mothers' feelings of guilt and shame at being in prison, their lack of control over their own environment, and their difficulties with prison authority figures which may be reminiscent of punitive parenting that they experienced in the past (Baradon et al., 2008).

On the other hand, the prison may mitigate some of the external threats to the early parent–infant relationship for some of these families. It is a stable and predictable environment where mothers and babies are protected from other risk factors such as domestic violence, substance misuse, homelessness, and poverty. Thus, for some mothers and babies, this period of co-residence may provide them with an opportunity to build their relationship in a context which is less chaotic than they may otherwise have experienced. Prison staff may become a source of support and stability for the mothers.

Controlled outcome studies are needed to understand the impact of the prison environment on the mother–infant relationship and the relative effects of attachment-based interventions.

### Attachment-related interventions for incarcerated mothers and their children

A number of interventions have recently been developed to support the relationship between incarcerated mothers and their children (Baradon et al., 2008; Byrne, Goshin, & Joestl, 2010; Cassidy, Poehlmann, & Shaver, 2010; Raikes, 2009; Shlafer & Poehlmann, 2010) and there is some evidence for their efficacy. Infants who remain with their mothers in custody and participate in relational interventions are just as likely to develop secure attachments as those in low-risk samples (Byrne et al., 2010; Cassidy et al., 2010). Their mothers are also likely to demonstrate levels of sensitivity comparable to mothers in a community comparison group (Cassidy et al., 2010) and improved levels of reflective functioning (Baradon et al., 2008). However, none of these studies included a control group and little is known about the outcomes for mothers and infants who remain together in a prison setting without any relationship-focused intervention.

### The New Beginnings program

New Beginnings is a manualized attachment-based intervention developed specifically for mothers and babies in prison (Baradon, 2006). Details of the development and content of the intervention have been reported elsewhere (Baradon et al., 2008). In a pilot evaluation (Baradon et al., 2008), mothers who participated in the program demonstrated a significant increase their Reflective Functioning capacity, the ability to think about their own internal states and those of their babies. A qualitative analysis gave insight to the areas of change in the mothers' representations of their relationship with their babies from pre- to post-intervention. Their defensive idealization gave way to a more complex, multi-dimensional depiction of themselves and their babies in relation to each other. They also demonstrated a shift towards understanding the baby as a person with a separate, and therefore different, mind. These capacities are associated with security of attachment and with more optimal parenting behavior (Slade et al., 2005).

The current study evaluates the outcomes of the intervention in a randomized controlled design. The aims of this larger trial were to determine whether these initial findings could be replicated, to determine whether there were any other changes in the quality of mother–infant relationship over time, and to compare the outcomes of the intervention with outcomes for mothers and babies not participating in the program.

## Methods

### Participants

**Figure 1.**
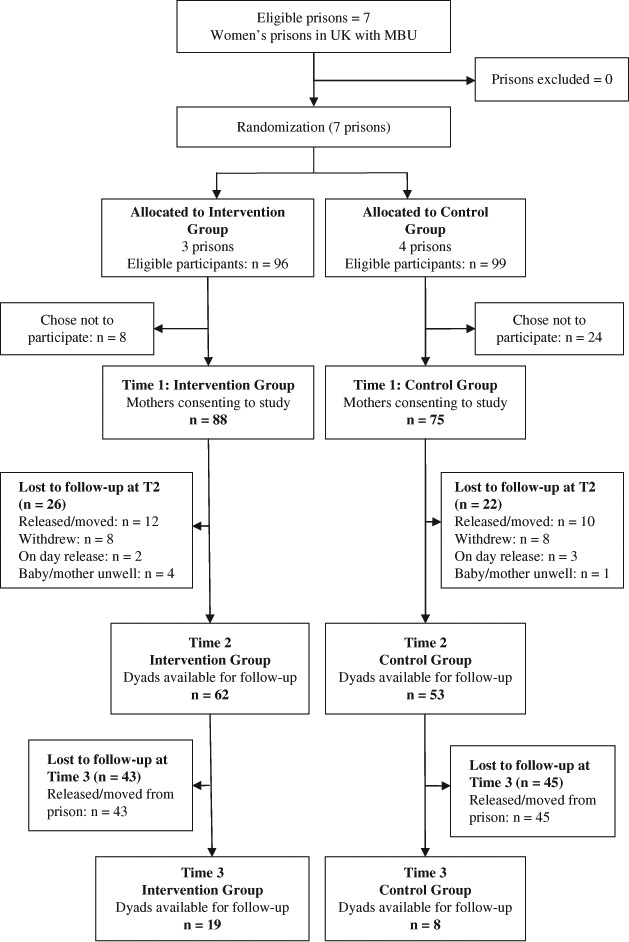
Consort diagram showing the flow of participants through the trial.

Mothers and babies were recruited from all seven MBUs. There were 88 dyads in the intervention prisons and 75 dyads in the control prisons who consented to participate in the study. The recruitment and retention rates are presented in the consort diagram (Figure 1). The attrition rates were high, with 29% and 83% of the sample being lost to follow up at Time 2 and Time 3, respectively. The majority of these dyads could not be followed up as they had been released or moved to a different prison. There were no significant differences between drop-outs and those who were followed-up in terms of maternal age (*t* = −0.6, ns), infant age (*t* = 0.7, ns), maternal ethnicity (*X*^2^ = 2.0, ns), infant ethnicity (*X*^2^ = 1.0, ns), infant gender (*X*^2^ = 1.2, ns), or baseline maternal depression scores (*t* = −0.5, ns). There were, however, differences between the groups in terms of maternal education; mothers with no formal qualifications were less likely to be seen at follow-up (*X*^2^ = 12.5, *p* = 0.01).

The demographic characteristics of the sample population are displayed in Table 1. There were no significant differences between the intervention and control groups in terms of maternal age and education, infant age and gender, or the number of other children the mother has had. There were however significant differences between the groups in terms of both maternal ethnicity, *X*^2^ (4, *N* = 163) = 12.5, *p* = 0.01, and infant ethnicity, *X*^2^ (4, *N* = 163) = 14.0, *p* = 0.07, with a larger proportion of white mothers and infants in the control group.

**Table 1.** Demographic data for the intervention and control samples.

	Control group *N* = 75			Intervention group *N* = 88			Intervention vs. controls	
	Range	Mean	*SD*	Range	Mean	*SD*	*t* (*df*)	*p*
Maternal age (years)	18–42	27.6	5.6	18–42	26.2	6.4	1.5 (160)	0.12
Infant age (months)	0.1–18.5	4.4	4.6	0.2–23.0	4.9	4.5	0.7 (161)	0.47

	*N*	%		*N*	%			

Maternal ethnicity							*X*^2^ (*df*)	*p*
White	51	68.0		38	43.2		12.5 (4)	0.01
Black	15	20.0		37	42.0			
Asian	4	5.3		6	6.8			
Mixed	4	5.3		7	8.0			
Other	1	1.3		0	0			
Infant ethnicity							*X*^2^ (*df*)	*p*
White	45	60.0		38	43.2		14.0 (4)	0.07
Black	11	14.7		34	38.6			
Asian	4	5.3		6	6.8			
Mixed	14	18.7		9	10.2			
Other	1	1.3		1	1.1			
Infant gender							*X*^2^ (*df*)	*p*
Male	30	40.0		34	38.6		0.03 (1)	0.87
Female	45	60.0		54	61.4			
Number of children							*X*^2^ (*df*)	*p*
First time mother	34	45.3		50	56.8		1.66 (1)	0.20
Other children	39	52.0		37	42.0			
Missing	2	2.7		1	1.1			
Level of education							*X*^2^ (*df*)	*p*
No qualifications	31	41.3		32	36.4		5.9 (3)	.12
Basic education	17	22.7		25	28.4			
Further education	20	26.7		18	20.5			
Higher education	1	1.3		8	9.1			
Missing	6	8.0		5	5.7			

### Ethical approval

This study was granted ethical approval by the University College London Research Ethics Committee.

### Inclusion and exclusion criteria

Mother–baby dyads staying on MBUs in the participating prisons during the recruitment period were eligible to take part in the study. Dyads were excluded if the mother was not sufficiently fluent in English to be able to give informed consent or take part in the research, or if she and her baby were known to be due for release before the first follow-up interview.

### Experimental design

A cluster randomized design was used, where prisons served as clusters and were randomly assigned to the intervention or control conditions. Although this was a cluster randomized trial, the unit of inference remained at the individual level (Campbell, Elbourne, & Altman, 2004). All seven women's prisons in the UK which had mother–baby units were eligible to participate in the research and all consented to do so. Randomization was carried out by an independent statistician. Three prisons were allocated to the intervention condition, where the New Beginnings programs took place, and four prisons were allocated the control condition where no New Beginnings courses took place.

Participating mother–baby dyads were assessed with an identical battery of measures at baseline, at the end of treatment, and again two months later. For the intervention group, the baseline time-point was before the start of the New Beginnings course and follow-up assessments took place at the end of the program five weeks later. For the comparison group, mother–infant dyads were assessed immediately after consenting to participate and again five weeks later. Mother–infant dyads in both groups who remained on the units were followed up two months after the second interview.

### Recruitment and procedure

During the recruitment phase, the research team liaised regularly with the mother–baby unit staff to ascertain the numbers of new mothers and babies entering the MBUs. As soon as there were sufficient numbers of new dyads in the intervention sites, a New Beginnings course was arranged and these mothers and babies were invited to participate in the intervention and research. In the control sites, the researcher paid regular visits to the prisons to recruit and interview new mothers who had entered the units. One week prior to the researcher visit to a MBU, eligible mothers were given information sheets about the study by MBU staff. The staff then arranged for the eligible and willing mothers and babies to meet with the research team. During the initial assessment visits, the researcher met with each mother and baby in a private room in the prison. During this meeting, the researcher read aloud the information sheet and consent form and time was given for questions before the mothers gave informed consent for participation. It was made clear to each potential participant that they were free to leave the research at any time and doing so would have no implications for their involvement on the New Beginnings course or their sentence.

After giving informed consent, the baseline assessments were carried out. During these assessments, mothers were interviewed, completed a set of questionnaires, and were filmed interacting with their babies. As a thank you for their time for the research, the mothers were given two photos at each assessment from the video-recorded interaction.

### Intervention

New Beginnings is a manualized attachment-based group intervention. Each program was carried out over eight sessions (two sessions of two hours each per day, one day per week) over a four week period. The groups were comprised of up to six mother–baby dyads and two parent–infant psychotherapists as facilitators. All sessions took place on a baby mat on the floor so that the babies were able to actively participate in the sessions.

Specific topics were covered through group discussion, handouts, individual worksheets, and homework tasks. The topics of each session were selected as potential triggers of the attachment relationship. These were explored through discussions of dedicated topics that link past and present patterns of relating, and by the careful observation of, and reflection on, non-conscious behaviors between mothers and babies. The facilitators and mothers in the group observed and noted the communications from the babies to their mothers, and the mothers' responses to their infants' signals. The purpose of group was also to help mothers to make links between their babies' behavior and their internal emotional world, to observe their own states of mind, and to think about how their own states of mind and those of their babies are separate but may also influence each other. The intervention has been described in more detail elsewhere (Baradon et al., 2008).

Seventeen New Beginnings courses were run as part of this trial, with 88 mother–infant dyads participating in the intervention during the study period. The mean number of clinical sessions attended was 7.1 (*SD* = 1.6). Most (87%) of the mothers and babies attended at least half of the sessions offered.

### Control condition

The New Beginnings courses were not held in the control prisons during the study period. The MBU units were otherwise very similar for both the intervention and control groups (HM Prison Service, 2008). Mothers and babies in both groups had access to standard health and social care provision as provided by the prison service.

### Measures

In addition to basic demographic information, the following standardized instruments were used in this study:

#### The Parent Development Interview (PDI; Slade, Aber, Bresgi, Berger, & Kaplan, 2004)

The PDI is a semi-structured interview used to explore the mother's perceptions of her child, her experience of being a parent, and how she perceives the relationship between herself and her baby. The interview also asks the mother to describe her own childhood experiences with her parents and how these impact on her current relationship with her child. The PDI was administered to mothers at baseline and at the first follow-up. These were transcribed verbatim and coded for Reflective Functioning (RF; Slade et al., 2004); the mother's capacity to accurately attribute and evaluate her own and her child's thoughts and feelings within their relationship. Overall Reflective Functioning scores for each interview range from −1 (low) to 9 (high). Reflective Functioning has been linked with parent–infant attachment, with higher RF scores associated with more securely attached relationships (Slade et al., 2005), and more optimal parenting behavior (Grienenberger, Kelly, & Slade, 2005).

The transcriptions of the interviews were coded by four fully trained and reliable coders who were blind to the treatment condition and time point (intervention or control group, baseline, or follow-up). A subset of 30 interview transcripts were coded by all raters and the inter-rater reliability for the overall RF score was good (ICC = 0.834).

#### The Center for Epidemiologic Studies Depression Scale (CES-D; Radloff 1977)

The CES-D scale is a 20-item self-report scale designed to measure depressive symptomatology in the general population. Each item is rated on a scale from 0 to 3 in terms of frequency of occurrence during the past week. The total score may range from 0 to 60, with a score of 16 or more indicating impairment. The reliability of the CES-D has been tested on clinic populations (Radloff, 1977) and on probability samples of US households (Radloff, 1977; Weissman, Sholomskas, Pottenger, Prusoff, & Locke, 1977). Results of these investigations indicated that the scale has high internal consistency reliability, acceptable test–retest stability, and good construct validity in both clinical and community samples.

#### The Mother's Object Relations Scales (MORS; Danis, Oates, & Gervai, 2005; Milford & Oates, 2009)

The MORS is a self-report measure for quantitatively assessing core features of mothers' internal working models of their infants. The short form is comprised of 14 items which are rated on a 6-point scale. The items fall into two dimensions of mothers' representations of their infant, “warmth” and “invasion”, each with a potential range of 0–42. Higher scores for warmth indicate more positive representations of the infant, while higher scores for invasion indicate more negative perceptions of the infant. The Warmth subscale includes items such as “My baby likes doing things with me”, and the Invasion subscale includes items such as “My baby irritates me”. The measure has demonstrated adequate internal consistency, test–retest reliability, and concurrent and discriminant validity (Oates & Gervai, submitted; Oates, Gervai, Danis, Lakatosb, & Davies, submitted).

#### Mother–infant interaction

In order to explore changes in the quality of the mothers' and infants' interactive behaviors, mothers and their infants were videotaped in free-play for 10 minutes. Mothers were asked to “be with your baby as you usually are”. These videorecorded interactions were coded using the Coding Interactive Behavior (CIB) scales (Feldman, 1998).

The CIB scales are comprised of 45 discrete items which are rated on a 5-point scale for the frequency and intensity that the behavior is observed (22 items relating to parental behavior, 16 items relating to the child's behavior, and 5 items relating to the quality of dyadic interaction as a whole). The CIB has shown variations and sensitivity to risk factors, such as maternal cocaine use (Mayes et al., 1997), delivery pain (Ferber & Feldman, 2005), and infant prematurity (Keren, Feldman, Eidelman, Sirota, & Lester, 2003) and sensitivity to change in studies of Kangaroo Care (Feldman, Weller, Sirota, & Eidelman, 2003) and massage therapy (Ferber et al., 2005) for preterm infants.

The authors suggest that the scales are aggregated into eight subscales based on the theoretical links between items. However, for this dataset the internal consistency for most of the recommended subscales was moderate to poor. For this reason, a principle components factor analysis with varimax rotation was carried out to summarize the coding scales. The resulting three-factor solution was used as the basis for the computation of three alternative subscales which resulted in greatly improved internal consistencies:

(1) Dyadic Attunement. The items in this subscale reflect an overall mutuality between parent and infant. The factor relates to an interaction where the parent is sensitive, non-intrusive, consistent, and supportive. There is no tension or constriction and the interaction is reciprocal and well adapted to the affective state of each partner. It is the sum of 11 items, with a potential range of 11–55 (Cronbach's α = 0.940).
(2) Parent Positive Engagement. This subscale relates to interactions where the parent looks and talks at her baby positively, does not appear depressed, and is enthusiastic in engaging with her baby. It is the sum of five items, with a potential range of 5–25 (Cronbach's α = 0.833).
(3) Child Involvement. This subscale incorporates most of the items relating to the infants' behavior. High scores reflect infants who are clearly positively involved in the interaction through gaze, vocalization, and initiation of mutual contact. It is the sum of six items, with a potential range of 6–30 (Cronbach's α = 0.857).

The videos were coded by four trained and reliable coders who were blind to both the group and time-point. A subset of 10 videos was rated by all coders, and the interrater reliabilities for each subscale were high: Dyadic Attunement (α = 0.905), Parent Positive Engagement (α = 0.957), and Child Involvement (α = 0.961).

### Statistical analysis

The adequacy of randomization was assessed by conducting between-group comparisons of baseline characteristics on all measures using chi-square for dichotomous variables and independent *t*-tests for interval data. Most of the outcome measures were normally distributed and were measured on continuous scales. The primary and secondary outcome measures at each time point were analyzed using general linear models. The analysis focused on changes between baseline and the end of treatment follow-up. The very small number of dyads who were available for the second follow-up did not allow for this data point to be reliably included in the analysis.

Some mothers chose not to participate in some parts of the assessment, so sample sizes vary for the different measures. The sample sizes are reported for each set of analyses.

As there was some sample attrition between the baseline and post-treatment follow up, mixed effects linear growth curve models were also estimated to confirm results in an intention-to-treat analysis which included all available data. Multiple imputation was used to estimate missing data for dyads who were followed up but who had incomplete data for some measures. Demographic covariates that could moderate or account for some of the group differences were included in the models and the results were compared with models without the covariates. SPSS version 19 and Stata version 10 were used throughout the statistical analysis.

## Results

### Reflective functioning on the Parent Development Interview

The primary outcome measure of the study was RF scores on the PDI between Time 1 and Time 2. The mean scores are displayed in Table 2.

**Table 2.** Outcomes for participants by treatment assignment.

	Baseline		Post-treatment	
	Control	Intervention	Control	Intervention
PDI RF ratings	*n* = 70	*n* = 80	*n* = 52	*n* = 57
Mean (*SD*)	3.59 (1.47)	3.18 (1.38)	3.15 (1.33)	3.54 (1.57)
Coding Interactive Behavior	*n* = 51	*n* = 67	*n* = 37	*n* = 51
Dyadic Attunement: Mean (*SD*)	41.63 (7.5)	38.15 (9.6)	38.06 (7.3)	34.98 (8.5)
Parent Positive Engagement: Mean (*SD*)	20.34 (2.9)	19.63 (3.8)	19.30 (3.2)	19.13 (2.7)
Child Involvement: Mean (*SD*)	16.34 (5.0)	16.85 (5.3)	16.99 (5.0)	16.62 (4.4)
CES-D	*n* = 70	*n* = 87	*n* = 53	*n* = 62
Total: Mean (*SD*)	15.3 (9.7)	13.9 (8.2)	15.3 (11.8)	13.6 (9.4)
Caseness: *N* (%)	35 (50%)	36 (41%)	25 (47%)	23 (37%)
MORS	*n* = 50	*n* = 49	*n* = 40	*n* = 31
Warmth: Mean (*SD*)	24.6 (7.3)	28.3 (6.5)	27.2 (5.6)	29.5 (4.6)
Invasion: Mean (*SD*)	7.3 (5.2)	7.0 (4.8)	8.3 (5.7)	7.7 (4.3)

A repeated measures analysis of variance was carried out to assess changes in RF for the two groups from pre- to post-intervention. Results showed that the interaction effect of treatment group and time was significant, *F*(1) = 12.341, *p* = 0.001, η_p_^2^ = 0.103. To confirm these results in an intention-to-treat analysis which includes all data, the mixed effects linear growth curve model was estimated (Table 3). The results indicate that the rating of RF assessed at follow-up increased significantly for the treatment group relative to the controls, expβk = 0.91; 95% CI: 0.3, 1.5; *p* = 0.002. The change in both groups over the time of the intervention was substantial, *t*(56) = −2.43, *p* = 0.02 and *t*(51) = 2.52, *p* = 0.02 for the intervention and control groups, respectively. This difference between the slopes reflects a significant decline in the group not receiving the intervention and a modest but statistically significant improvement in the intervention group. Although infant age was significantly correlated with RF at both time points, it was not a significant covariate in the general linear model or mixed effects regression analyses.

**Table 3.** Results of multilevel random linear regression for RF and mother–infant interaction ratings.

	Rate of change (slope) of individual trajectory (expβk) for pre- vs. post-tests				
			Change over time (95% CI)		Group effect over time (95% CI)	
	Wald Statistic χ2 (*df* = 3)	*p*<	Coefficient	*p*<	Coefficient	*p*<
PDI RF ratings	23.9	0.000	−0.48 (−0.9, −0.1)	0.020	0.91 (0.3, 1.5)	0.002
CIB Dyadic Attunement	32.65	0.000	−6.92 (−10.22; −3.62)	0.000	3.81 (.13; 7.50)	0.043
CIB Parent Positive Engagement	15.38	0.009	−2.30 (−3.62; −.99)	0.001	1.36 (−.10; 2.83)	0.068
CIB Child Involvement	1.94	ns	.11 (−1.67; 1.88)	ns	−0.21 (−2.19; 1.77)	ns

### Observation of mother–infant interaction: Outcomes for quality of behavioral interaction

A repeated measures multivariate analysis of covariance (MANCOVA) was conducted to assess changes in the treatment groups over time on the three CIB subscales. The independent variable was the treatment group and the three subscales of the CIB served as the dependent variables in the analysis. The number of children each mother had was significantly correlated with two of the three CIB subscales at follow-up: Dyadic Attunement (*r* = 0.373, *p* = 0.002) and Parent Positive Engagement (*r* = 0.385, *p* = 0.002), and was therefore included as a covariate in the analysis. The a priori level of significance was set at .05. The Wilks' Λ results showed a significant main effect of time, *F*(3) = 2.455, *p* = 0.000, η^2^_*p*_ = 0.273, but the interaction between group and time was only marginally significant, *F*(3) = 2.455, *p* = 0.072, η^2^_*p*_ = 0.111. Tests of within subjects contrasts for the subscales showed that the time by group interaction was significant for Dyadic Attunement and Parent Positive Engagement, *F*(1) = 5.594, *p* = 0.021, η^2^_*p*_ = 0.084 and *F*(1) = 4.617, *p* = 0.036, η^2^_*p*_ = 0.070, respectively, but not Child Involvement, *F*(1) = 0.066, ns, η^2^_*p*_ = 0.001.

As the CIB scales relating to parental and overall dyadic behavior demonstrated different effects for the groups over time than the measure of infant behavior, the MANCOVA was repeated with only the Dyadic Attunement and Parent Positive Engagement subscales as dependent variables. The Wilks' Λ results showed a significant main effect of time, *F*(3) = 9.516, *p* = 0.000, η^2^_*p*_ = 0.241, and the interaction between group and time was significant, *F*(3) = 3.387, *p* = 0.040, η^2^_*p*_ = 0.101.

A series of random regression models were conducted to verify these results in an intent-to-treat analysis, making use of all available data (Table 3). For Dyadic Attunement, the mixed effects regression model showed a significant group by time interaction effect, *expβk* = 3.81; 95% CI: 0.13, 7.50; *z* = 2.03; *p* = 0.043. Dyads in the control group demonstrated greater dyadic attunement at baseline than those in the intervention group, but these dyads deteriorated on this measure over time while the dyadic attunement for intervention group dyads remained relatively stable over time. The interaction between group and time on Parent Positive Engagement was marginally significant, *expβk* = 1.36; 95% CI: −0.10, 2.83; *z* = 1.82; *p* = 0.068, but the confidence intervals indicated that this may not be a reliable model. The mixed effects regression model for Child Involvement was not significant, *Wald* χ^2^ = 1.94, ns, indicating no effect of time or time by group interaction on this subscale.

### Association between RF and mother–infant interaction

RF and mother–infant interaction scores followed similar patterns of change over time for the two groups. A mediation model was considered whereby change in maternal RF mediated the differential change in interactions for the two groups, or vice versa. As shown in Table 4, RF at both time points was moderately associated with maternal positive engagement and child involvement in the parent–infant interactions. However, the changes in RF and interaction scores were not significantly correlated. Thus, the conditions for testing the mediation model were not met (Baron & Kenny, 1986) and changes on these measures were independent of each other.

**Table 4.** Correlations between RF and mother–infant interaction ratings at baseline (T1), follow-up (T2), and change over time.

	RF T1	RF T2	RF change
Dyadic Attunement T1	0.059	0.095	0.000
Dyadic Attunement T2	0.159	0.173	0.036
Dyadic Attunement change	0.078	0.005	−0.042
Parent Positive Engagement T1	0.232*	0.211	−0.058
Parent Positive Engagement T2	0.187	0.195	−0.001
Parent Positive Engagement change	−0.111	0.013	0.075
Child Involvement T1	0.209*	0.332**	0.170
Child Involvement T2	0.204	0.282*	0.058
Child Involvement change	0.022	−.111	−0.126

* Correlation is significant at the 0.05 level (2-tailed)

** Correlation is significant at the 0.01 level (2-tailed)

### Maternal depression

The CES-D scores are presented in Table 2. A repeated measures analysis of variance was carried out to analyze changes in self-reported depression on the CES-D for the two groups from baseline to the end of treatment follow-up. Neither the main effect of depression over time nor the interaction between depression levels and intervention group were significant, *F*(1) = 0.089, ns, and *F*(1) = 0.027, ns, respectively. These results were stable when multiple imputation methods were used to replace missing data for those followed up at this time point.

### Maternal object representations

The means and standard deviations for the warmth and invasion scales of the MORS are displayed in Table 2. A repeated measures analysis of variance demonstrated a marked increase in self-reported warmth in both groups which was statistically significant. Mothers in both groups demonstrated increased feelings of warmth towards their infants over time, *F*(1) = 10.333, *p* = 0.002, η_*p*_^2^ = 0.147. The interaction between treatment group and MORS warmth scores was not significant *F*(1) = 1.446, ns. Both infant age and maternal ethnicity were significantly correlated with the MORS warmth scores at both time points. The inclusion of either or both of these measures as covariates did not alter the main or interaction effects.

There were no significant findings related to the second of the MORS scales, invasiveness. Both the main effect of invasion over time and the interaction between invasion scores and intervention group were non-significant, *F*(1) = 0.455, ns, and *F*(1) = 0.275, ns, respectively.

These results were stable when multiple imputation was used to replace missing data for cases who were followed-up but who did not complete the questionnaire.

## Discussion

This study is one of the first to look at intervention outcomes for parents and infants co-residing in prison in a randomized controlled design. At the outset, the dyads presented with non-optimal outcomes on most measures. Mothers tended to have very low levels of reflective functioning when talking about their relationship with their babies which were comparable to levels seen in other high-risk samples (Schechter et al., 2008; Suchman et al., 2010). The quality of parent–infant interactions for this group also tended to be relatively poor, even when compared with a community sample of mothers with mental health difficulties and their infants who had been referred for parent–infant psychotherapy (Gale, 2008). Furthermore, almost half of the mothers in this sample reported clinically significant levels of depression. All of these factors have been implicated in the development of insecure/disorganized attachment relationships and poor outcomes for the child (Grienenberger et al., 2005; Lyons-Ruth, 1992; Slade et al., 2005), highlighting the importance of research and intervention programs targeting this population.

### Changes in maternal RF and parent–infant behavior

There were two relatively independent improvements that were observed to be associated with the New Beginnings program. Maternal RF and the quality of maternal/dyadic behavior showed a general deterioration over time in the control sample, and this decline in both domains appeared to be moderated by the intervention.

The deterioration in RF and interaction scores for the control group was unexpected and incongruous to other studies which have reported relative stability of maternal sensitivity and RF in community control samples (Kemppinen, Kumpulainen, Raita-Hasu, Moilanen, & Ebeling, 2006; Suchman, Decoste, McMahon, Rounsaville, & Mayes, 2011; Xue, Moran, Pederson, & Bento, 2010). There may be a complex interplay of factors specific to this sample which account for these findings. The prison environment may, in some cases, precipitate further risks to the mother–baby relationship. For example, some mothers on the MBU's will be separated from their infants later on and, without adequate preparation, may become gradually less engaged with their baby as the time of separation draws nearer. Furthermore, the separation of the mother and baby from their family and social networks in the confined prison setting may have detrimental consequences for the quality of their relationship. Although the intensity of the relationship between mother and baby in the prison environment may facilitate the development of a close and secure attachment bond (Goshin, 2010; Goshin & Byrne, 2009), it may paradoxically also heighten and distort some of the less adaptive characteristics of their relationship. For example, qualitative analyses of the interviews with these mothers have demonstrated a great deal of role reversal in the mothers' representations of their babies (Baradon et al., 2008; Sleed, 2011). Many mothers identified their infants as their source of comfort and as someone who helped them to survive the difficulty of being in prison. The interviews with some mothers revealed a parentification of the infant or a spousal-like representation of the mother–baby relationship. As mothers spend more time in prison and away from their usual adult support network, their babies may be increasingly represented as the person who provides emotional regulation for them. Role reversal, as observed in parental representations and behavior, has been highlighted as a key risk factor for the development of disorganized attachment relationships (George & Solomon, 2008; Lyons-Ruth, 2003). Other dominant themes and indicators of risk from the interviews with the mothers were the overwhelming feelings of guilt and hostility (Baradon et al., 2008), and these too may become more entrenched and infant-directed over time if adequate provision is not made to intervene in this process.

The findings of this study do not imply that babies who remain with their mothers in prison necessarily fare worse than they would have had they been separated. On the contrary, there is evidence that children who are separated from their mothers due to incarceration have relatively worse outcomes than those who remain with their mothers in prison, although both groups have elevated aggressive behavioral problems in the preschool years (Goshin, 2010). Our findings, taken in the context of this longitudinal research, suggest that even when mothers and babies remain together, they represent a high-risk population that may benefit from relational intervention.

Importantly, this study showed that the New Beginnings program may moderate the potential deterioration of the quality of the parent–infant relationship for these families over time. The intervention gives the mothers the opportunity to focus on their own and their infants' behaviors and states of mind in relation to each other, shifting their understanding of their babies' experiences to a representational level and strengthening the mothers' capacity for mentalization. This is often done in the context of thinking about early childhood trauma and how feelings about these experiences are aroused by the prison setting and by their baby. The course focuses the mothers' minds on their babies' internal states, their infantile states of dependency, and their natural ways of communicating. Curiosity is an inherent ingredient to the course and all of the questions asked model interest, empathy, perspective-taking, and assume that there are reasons for behaviors. The group nature of the intervention in a setting where the mothers and babies co-reside may be essential to the potency of the relatively brief intervention as the mothers may continue to model and reinforce the mentalizing stance with each other outside of the sessions (Sleed, James, Baradon, Newbery, & Fonagy, 2012).

The findings of this study shed some light on the specific components of the parent–infant relationship that shift as a result of the intervention. Firstly, of the three behavioral rating subscales, it was the quality of overall dyadic behavior and maternal behavior that improved for the intervention group relative to the control dyads, not the degree of infant involvement. Thus, the program appears to initially be more effective in changing the way the mothers think about and interact with their babies than changing the infants' interactive involvement. It is likely that the follow-up period for the study was too brief to pick up on any changes in the babies' level of responsiveness that might have occurred. Further longitudinal research is needed to test the stability of results of this study and understand the long-term implications for the babies. However, it is likely that over time the babies will change their behavior in response to the changes in how their mothers think about and interact with them, rendering them more likely to develop secure attachment bonds (De Wolff & van IJzendoorn, 1997; Grienenberger et al., 2005; Slade et al., 2005).

Another interesting finding was that the observed changes in maternal RF and behavior for each of the groups were independent of each other. We are therefore unable to determine whether one of these domains may be modifying the other. We did find significant associations between maternal RF and the quality of interactive behavior, as have other studies (Grienenberger et al., 2005). However, it was the degree of change in each of these outcomes which was unrelated, suggesting that there might be a differential rate of change for each of them. It might be that behavioral changes occur more slowly and subtly than changes in the mothers' capacity for mentalization, or vice versa. Nevertheless, we have an independent modeling effect in these two measures over time. Until we have more information about the longer-term relationship between these two aspects of parent–infant functioning for this population, interventions should target both the representational and the behavioral level. Both of these aspects of the relationship are important precursors for attachment security and positive long-term outcomes for the infants (Belsky & Fearon, 2002; De Wolff & van IJzendoorn, 1997; Slade et al., 2005).

### Changes in maternal depression and object relationships

No intervention effects were found on the two self-report questionnaires relating to mothers' depression and representations of their infants. The fact that there were no differential changes in maternal depression for the two groups indicates that it was not changes in the mothers' emotional state which moderated the mentalization and behavioral effects that were found. This finding suggests that, at least for this population, difficulties in the mother–infant relationship are not likely to be alleviated through the treatment of maternal depression alone. This provides some validation for dyadic rather than maternal-focused interventions for improving relational outcomes for these parents and babies.

The fact that no treatment effects were found on the MORS was unexpected. Both independently rated measures of dyadic functioning showed similar changes over time for each of the groups, but these did not correspond to changes in the content of mothers' self-reported representations of their babies. This may be indicative of a failure of the parent-report method in this sample, possibly because these mothers are more likely to give socially desirable responses to statements that may feel threatening, such as “My baby irritates me”. The high level of idealization that was apparent in these mothers' responses to questions on the PDI lends support to this explanation (Baradon et al., 2008). Other studies with incarcerated mothers and their children have reported similar biases in mother-report questionnaires (Goshin, 2010). Thus, in-depth independent assessments of parent–infant relational functioning are likely to be more valid instruments for use with this population.

### Limitations

There are some limitations to this study which should be taken into account. Firstly, the intervention and control groups were not perfectly matched at baseline. This is likely to be the result of cluster randomization. The intervention prisons were geographically closer to the international airports and therefore may have included an over-representative number of non-national mothers and babies, leading to ethnic and language differences between the two groups. Mothers in the control group tended to have higher levels of RF and better quality interactions with their infants at baseline than those in the intervention group. The findings of this study may therefore reflect a regression to the mean rather than intervention effects. Cluster randomized trials are prone to methodological biases, including the possibility of baseline differences between groups (Hahn, Puffer, Torgerson, & Watson, 2005). Given the residential nature of MBU's and the group format of the intervention, individual allocation was not possible.

The second major shortcoming of this research was the high rate of attrition. This problem is not uncommon in intervention studies of incarcerated women and their children (Cassidy et al., 2010; Shlafer, Poehlmann, Coffino, & Hanneman, 2009). One of the major reasons for the high rate of attrition was the very rapid turnover of women in the units. Many women prisoners in the United Kingdom serve brief sentences, with more than half being sentenced to custody for six months or less (Ministry of Justice, 2010). Although precautions were taken to exclude dyads who were due for release before the first follow-up, many women were on remand or unsentenced at the time of recruitment and were enrolled but released before they could be seen for the follow-up.

The obstacles to conducting research in correctional facilities may be one of the reasons for the relative poverty of evaluated interventions for incarcerated parents and their children (Bretherton, 2010). Despite these methodological shortcomings, this study is one of the first to explore the outcomes for mothers and babies in prison with and without an attachment-focused intervention. Future research which looks at long-term outcomes after the mothers and babies are released from prison will broaden our understanding of the stability of the findings of this study.

## Conclusion

The findings of this study have highlighted the high risk nature of this group of mothers and babies and the poor trajectory for many of them. A relationship-focused group intervention may be an effective means of helping these mothers and infants to strengthen their early attachment bond and offset some of the deleterious outcomes that have been reported for children of incarcerated parents. This study has supported what has been known since the mid-1990s: that relatively brief, focused interventions can be highly effective in shifting the quality of early parent–infant attachment relationships (van den Boom, 1994; van IJzendoorn, Juffer, & Duyvesteyn, 1995). The benefits of such short-term interventions are likely to be far-reaching and enduring (van den Boom, 1995). Taken together, these outcome studies suggest that there may be a period in the first year after having a baby when the mother's attachment and caregiving systems are reorganized and are particularly open to change. Brief interventions with high-risk dyads can capitalize on this window of opportunity.
